# Comprehensive clinical implementation, workflow, and FMEA of bespoke silicone bolus cast from 3D printed molds using open‐source resources

**DOI:** 10.1002/acm2.14498

**Published:** 2024-08-27

**Authors:** Dean Hobbis, Michael D. Armstrong, Samir H. Patel, Riley C. Tegtmeier, Brady S. Laughlin, Shadi Chitsazzadeh, Edward L. Clouser, Jennifer L. Smetanick, Justin Pettit, Justin D. Gagneur, Joshua B. Stoker, Yi Rong, Courtney R. Buckey

**Affiliations:** ^1^ Department of Radiation Oncology Mayo Clinic Arizona Phoenix Arizona USA; ^2^ Department of Radiation Oncology Washington University School of Medicine in St Louis St Louis Missouri USA; ^3^ University of South Florida Morsani College of Medicine and Tampa General Hospital Cancer Institute

**Keywords:** electrons, external beam, process map, quality assurance, risk analysis

## Abstract

**Background:**

Bolus materials have been used for decades in radiotherapy. Most frequently, these materials are utilized to bring dose closer to the skin surface to cover superficial targets optimally. While cavity filling, such as nasal cavities, is desirable, traditional commercial bolus is lacking, requiring other solutions. Recently, investigators have worked on utilizing 3D printing technology, including commercially available solutions, which can overcome some challenges with traditional bolus.

**Purpose:**

To utilize failure modes and effects analysis (FMEA) to successfully implement a comprehensive 3D printed bolus solution to replace commercial bolus in our clinic using a series of open‐source (or free) software products.

**Methods:**

3D printed molds for bespoke bolus were created by exporting the DICOM structures of the bolus designed in the treatment planning system and manipulated to create a multipart mold for 3D printing. A silicone (Ecoflex 00–30) mixture is poured into the mold and cured to form the bolus. Molds for sheet bolus of five thicknesses were also created. A comprehensive FMEA was performed to guide workflow adjustments and QA steps.

**Results:**

The process map identified 39 and 30 distinct steps for the bespoke and flat sheet bolus workflows, respectively. The corresponding FMEA highlighted 119 and 86 failure modes, with 69 shared between the processes. Misunderstanding of plan intent was a potential cause for most of the highest‐scoring failure modes, indicating that physics and dosimetry involvement early in the process is paramount.

**Conclusion:**

FMEA informed the design and implementation of QA steps to guarantee a safe and high‐quality comprehensive implementation of silicone bolus from 3D printed molds. This approach allows for greater adaptability not afforded by traditional bolus, as well as potential dissemination to other clinics due to the open‐source nature of the workflow.

## 1 INTRODUCTION

Three‐dimensional (3D) printing is a digital fabrication technology that utilizes an additive manufacturing approach to create physical objects with simple or complex geometries.^1^, ^2^, ^3^ The methods for 3D printing are characterized into seven distinct groups: binding jetting, directed energy deposition, material extrusion, material jetting, powder bed fusion, sheet lamination, and vat polymerization.^2^ Each method has targeted applications and material classes optimized for their use, making 3D printing and additive manufacturing versatile techniques used across many disciplines.^2^, ^4^, ^5^, ^6^, ^7^, ^8^, ^9^ Improved accessibility to 3D printing and complex geometries that can be achieved has led to an increased investigation in many fields, including medicine.^2^, ^4^, ^5^, ^6^, ^7^, ^8^, ^9^ Cost‐efficiency and highly customizable features make 3D printing of keen interest in radiation oncology applications, with quality assurance phantoms,^10^ brachytherapy applicators,^11^, ^12^, ^13^, ^14^ patient immobilization,^15^, ^16^, ^17^, ^18^ and bolus being investigated.^1^, ^3^, ^19^, ^20^, ^21^, ^22^, ^23^, ^24^, ^25^, ^26^

The fundamental physical properties of megavoltage electron beams (6–20 MeV) mean this therapy modality is limited to treating superficial target volumes extending ⪅ 6 cm from the patient's surface. The general aim for these treatments is to have the 90% isodose line covering the most distal surface of the planning target volume, resulting in ∼10% dose variation within the target volume. However, oblique incidence and heterogeneities significantly impact the dose uniformity of megavoltage electron beams. This can be of particular challenge in the head and neck region with complex surfaces, air cavities, and surrounding organs at risk. Moreover, the skin‐sparing effect of megavoltage photon beams means that superficial dose coverage can be challenging, often mitigated by using a buildup bolus to pull the isodose lines more superficially. Generally, the commercial bolus used in most clinics is standardized in sheets of uniform thickness with pre‐set increments. Several challenges are faced by commercially available bolus materials, such as mechanical stiffness, uniform thickness, and being overly adhesive, making it difficult for such sheets to conform to irregularly shaped or complex surfaces, fill cavities, or have a variable thickness. Quality assurance (QA) failures for commercial bolus, such as thickness and density variation, are, in our experience, not uncommon. These shortcomings can decrease the day‐to‐day reproducibility of bolus application and setup, possibly leading to air gaps and setup uncertainties that can adversely impact dosimetry.^27^, ^28^, ^29^ The decision to use a bolus can occur before or after the patient's computed tomography (CT) simulation scan, meaning the bolus may be added virtually in the treatment planning software (TPS) and would not be present on the patient at the time of simulation. This leads to additional challenges when applying a sheet of commercial bolus to conform to the patient and match the virtual bolus created on the TPS. Such challenges are particularly apparent in the head and neck region, where the nose, ears, and chin provide complex surfaces that the mechanical properties of traditional bolus material struggle to adhere to.^21^ 3D printing allows customizable bolus that can be printed to conform to individual patient's anatomy based on their CT scan, reducing uncertainty for daily setup variations and minimizing air gaps.^1^, ^3^, ^19^, ^20^, ^21^, ^22^, ^23^, ^24^, ^25^, ^26^ Moreover, the capability of 3D‐printing to create complex geometries means that a bolus can be designed for both buildup and tissue‐compensation, filling the nasal cavity or outer ear canal for example. The adaptability of being able to create complex bolus geometries virtually can result in reduced time and effort at the time of simulation. 3D printing via material extrusion, particularly fused deposition modeling (FDM), is the most commonly employed method due to its low cost and capability to print objects using more than one material or color.^30^ In FDM, a digital model of a 3D object is generated and processed into a series of thin layers, which are realized into a physical object by a 3D printer. The material filament is heated to a semi‐liquid state before the nozzle deposits ultra‐fine beads along the desired path, sequentially adding material layer by layer.^30^ Traditional 3D printing materials used in FDM, such as polylactic acid (PLA) and acylonite butadiene styrene (ABS), belong to the thermoplastic material family, meaning that even bolus custom‐printed for a patient's anatomy can be challenging due to the inherent rigidity of the material.^1^, ^3^, ^19^, ^30^ Moreover, material hardness has an impact on patient comfort. Thus, several alternative materials with better malleability have been investigated, such as Agilus and Tango.^19^, ^31^ Due to the inherent limitations of the mechanical properties of the 3D printable materials described above, as well as the high cost of the specialty printer required for Agilus and Tango, recent studies have explored silicone as a more optimal alternative.^21^, ^26^ Silicone is desirable due to its composition, flexibility, and softness, making it an ideal candidate for patient comfort, set up conformity, and reproducibility.^21^, ^26^ However, directly 3D printing silicone via extrusion is challenging, thus an approach that uses a 3D printed mold as a cast for poured silicone was utilized.^21^, ^26^

A systematic review by Rooney et al.^1^ highlights the lack of studies describing the effective use of and workflow systems for incorporating 3D printing applications into routine clinical practice, an area they describe as being pivotal to the widespread dissemination of this technology.^13^, ^18^, ^32^, ^33^ Chiu et al.^21^ discuss the clinical workflow for implementing such a bolus, however, AAPM Task Group 100 states that many errors that occur in radiation oncology are due to workflow and processes, not failures in devices and software, requiring a deeper investigation.^34^ Failure mode and effects analysis (FMEA) is a prospective tool that aims to provide a systematic understanding of the clinical impact and likelihood of failures for a given process. Process mapping combined with FMEA is crucial to help provide the information required to optimize quality and safety in patient care while assigning resources as efficiently as possible, particularly for new ventures. Thus, quality management (QM) methods such as process mapping and FMEA on new technology are paramount to their successful clinical implementation, as well as providing a comprehensive overview that is key to the dissemination of these processes to other insitutions.^1^, ^34^

The challenges associated with commercial bolus and the less‐than‐ideal mechanical properties of typical 3D printing materials led to our investigation into a more viable alternative. This study presents the commissioning and multi‐phase clinical implementation of bespoke silicone bolus and pre‐formed bolus sheets cast from 3D‐printed molds, virtually eliminating commercial bolus use in our clinic. Moreover, we present quality control (QC) and quality assurance (QA) recommendations for this workflow based on process mapping and FMEA, which can help bridge the gaps in the current reported literature.

## 2 MATERIALS AND METHODS

### 2.1 Mold creation

CT images and associated TPS‐designed bolus structures are exported in DICOM format from Eclipse (Varian Medical Systems, USA) and loaded into 3D Slicer (https://www.slicer.org/) to be converted to a mesh (STL file). An STL file containing the designed bolus is loaded into Meshmixer (https://meshmixer.com/) and manipulated to create a multi‐part mold (Figure S1). The mold is printed on one of several available printers, most often an Ultimaker S5 3D printer with a nozzle size of 0.8 mm using 2.85 mm PLA filament. The printed mold parts are assembled and sealed using a hot glue gun. Spring clamps augment the adhesion and are applied around the edges of the walls (Figure S2). After the sides are complete, a bead of hot melt glue is applied to the bottom and pressed onto a textured piece of reusable ABS plastic. The texture and the mold's weight ensure an easily removable, silicone‐tight bottom seal. Demolding is as simple as removing the clamps and walls, and pulling the two mold pieces apart.

### 2.2 Bolus fabrication

Ecoflex 00−30 from Smooth‐On was identified for the bespoke bolus material. All materials from Smooth‐On are independently verified to be skin‐safe, meaning the material shows no evidence of causing dermal contact sensitization and is classified as a non‐irritant.^35^ The two components of Ecoflex 00−30 are mixed in a 1:1 ratio in a disposable plastic mixing bowl with a total volume of 10% greater than needed to account for waste. Silicone Thinner is added at +10% by mass and thoroughly mixed for 3 min. The mixture is then degassed in a 5‐gallon vacuum container until the bubbling stops. The degassed mixture is poured into the mold using a funnel and cured for four hours. Adding Silicone Thinner lowers the ultimate hardness and stiffness of the cured material. At the same time, it also increases the pot working time, reduces viscosity for better flow over intricate details in the mold, and permits more rapid de‐gassing under vacuum. This type of bolus will be referred to as bespoke bolus (BB) for the remainder of the paper. The pre‐formed sheet bolus cast from 3D printed molds was made as above, with the addition of Silc Pig Electric Fluorescent Silicone Pigments (+3% by mass). The addition of colored pigmentation was to color code the five available thicknesses (0.3, 0.5, 0.7, 1.0, 1.2, and 1.5 cm), with each mold having a corresponding color (Figure S3) and will be referred to as color sheet bolus (CSB) for the remainder of this study. A detailed step‐by‐step process of mold creation and bolus fabrication can be found in the Supplemental Information.

### 2.3 Bolus quality assurance

The Shore value, a measure of hardness,^36^ was evaluated using a factory‐calibrated durometer (Rex Durometers, Model 1600‐00) for five pieces of BB. CT‐based QA was performed on a Siemens Somatom—Definition AS (Siemens Medical Solutions, USA) to ensure fidelity to the planned design. All boluses were CT scanned after fabrication and removal from the mold to evaluate HU values, appropriate thickness, shape fidelity, and signal homogeneity. CT Hounsfield units (HU) were investigated for test boluses to pre‐determine the HU values to be entered into the TPS before patient use. Five independent sheets of BB and five independent sheets of CSB spanning five different thicknesses were utilized for determination. Sheets of uniform thickness of the BB were deemed more suitable for HU determination than the complex geometry of the BB. The sheets were placed flat on top of the couch and scanned using an internal scanning protocol with a suitable FOV and consistent kV and mAs settings. Both longitudinal and transverse line profiles were taken for each bolus, with the longitudinal profile through the center of the bolus utilized to derive the average HU value.

Five independent BB and CSB boluses were fabricated to validate thickness. The CSB boluses of varying thickness were measured as above, laid flat on the CT couch, and scanned. The BB boluses were scanned while draped over the inner shell of the 3D‐printed mold. The scans were imported into Eclipse, and thickness measurements were taken at several locations normal to the 3D‐printed inner shell or couch surface using the measure distance tool.

Dosimetric validation was investigated using Gafchromic EBT‐3 film (Ashland, USA) for 0.5 cm thick flat sheets of SuperFlab, BB, and CSB bolus on a single Varian TrueBeam (Varian Medical Systems, USA) at our institution. The one‐scan protocol was utilized, with one un‐irradiated standard and two standards irradiated under reference conditions in a 6 MV beam (SSD = 100 cm, d = 1.5 cm, MU = 100, FS = 10 cm × 10 cm) was used. From reference conditions, the top 1.5 cm of solid water was removed and replaced by the 0.5 cm sheets of bolus. The EBT‐3 film was placed directly underneath the bolus at 0.5 cm depth, with 100 cm SSD to the surface of the bolus, 10 cm × 10 cm field size, and irradiated using 6 MV photons and 6 MeV electrons. Each measurement was repeated three times. The measured doses were compared to the expected doses calculated in the Eclipse TPS using the Anisotropic Analytical Algorithm (AAA, v15.6.06) and Electron Monte Carlo (EMC, v15.6.06) for photons and electrons, respectively. The HU values used for the calculations for comparison were from CT evaluation and the clinically utilized HU values. This study has received Institutional Review Board approval (IRB#: 23−000082).

### 2.4 Risk analysis methodology

The interdisciplinary nature of the modern radiation oncology practice necessitates the involvement of stakeholders from all teams (e.g., therapists, physicists, physicians, dosimetry, etc.) when new technology and procedures are being implemented. Each team member's unique perspective and expertise provide valuable insight into the process steps and failure modes. Such a team approach is critical in the safe and high‐quality implementation of new processes, allowing a more comprehensive risk assessment.

A process map (or tree or chart) is a prospective QM tool that illustrates the individual steps of a process while providing context on the temporal and physical relationships between them.^34^ The process map provides a chronological overview of the workflow, with the significant sub‐processes emerging from the main trunk, each with its own more detailed “branch” processes.^34^ It is essential that the level of detail of a process map is not too crude to hide crucial steps yet not so detailed to obscure the flow. Developing a process tree provides the foundation for performing FMEA. FMEA is a prospective tool that aims to assess the probability of failures at each step in the process map by identifying the possible failure modes and the consequential scoring of each identified failure. Each failure mode is scored based on the occurrence (*O*), i.e., how likely it is for a failure mode to occur, the severity (*S*) of the outcome as a result of the failure mode, and how likely the failure mode is to be detected in time to prevent an event or the lack of detectability (*D*). The product of these three metrics results in a single risk priority number (*RPN*) metric, *RPN* = *O·S·D*. A team approach was utilized to score failure modes. A second RPN metric was also computed using a lack of detectability score with a QA step present (*D_QA_*). The two team members (from physics, engineering, physician, and medical physics assistant teams) deemed to have the most intimate knowledge of a given step were tasked with scoring those failure modes based on Table 2 in AAPM's TG100.^34^ The individually scored failure modes were then combined by taking the average of the *O*, *S*, *D*, and *RPN* values. Following the completion of FMEA, the next step is to use this data to guide the development of a resource‐efficient and risk‐based QM program. This QM program will be adopted for phased clinical implementation to re‐evaluate the approach based on stakeholder feedback and the department's incident learning system.

## 3 RESULTS

All Shore values fell within the range of 18‐22 in testing for the BB with a standard deviation (2σ) of 2.7. Upon standardization of the mixing process, the BB and CSB boluses had an average HU value of 160 and 140, respectively. A small amount of “edge effect” —whereby the top and bottom surface give slightly higher HU values than the center—was observed in the transverse profiles for the CT HU evaluation. This would have a greater impact on thinner boluses and is considered in the average HU determination. The CT‐derived HU values for SuperFlab (250 HU) and CSB (140 HU) resulted in dose discrepancies of −1.0% and −2.1% for photons, respectively. The CT‐derived HU value for BB is 160 HU. Based on this data, a standardized value of 160 HU was chosen for BB and CSB clinical use. For photons, the clinically utilized HU values indicated a 2.6%, −3.6%, and −2.3% dose discrepancy between the measured and planned dose values for SuperFlab (0 HU), BB (160 HU), and CSB (160 HU), respectively. The electron dose discrepancies for the clinically used HU values for SuperFlab, BB, and CSB were −0.5%, 0.7%, and 0.6%, respectively. The dose difference for the CT‐derived HU values for electrons was 0.7% for both SuperFlab and CSB. The average measured thickness discrepancy for the BB and CSB boluses was 0.1 mm, with a maximum value of 0.2 mm. All silicone cast boluses were measured as the designed thickness within the measurement limitation of the tool and user.

**FIGURE 1:**
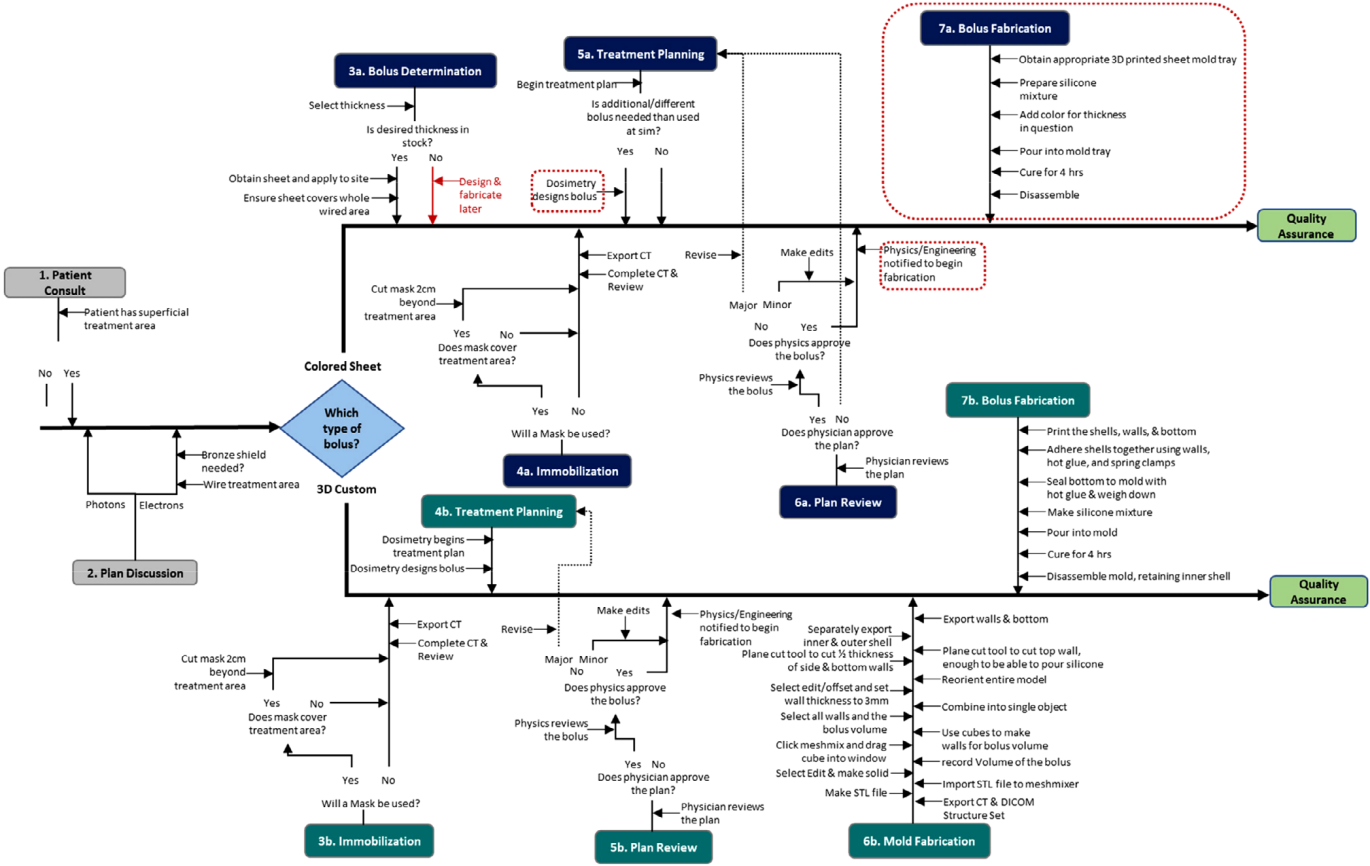
Process map for colored flat sheet (top) and bespoke (bottom) silicone bolus workflows, from patient consult through bolus quality assurance.

The process map for the BB and CSB is shown in Figure 1. The process map begins with the patient consult and ends with the completion of bolus fabrication before QA of the bolus. The BB workflow has 39 distinct steps, while the CSB has 30. The FMEA identified 119 and 86 failure modes for the BB and CSB processes, respectively. Of these, there are 69 shared failure modes for the two methods. The top 10 average RPN scores are shown in Table 1, while Table 2 shows the top 5 RPN scores that relate specifically to bolus creation/fabrication steps. The BB and CSB workflow had a maximum RPN of 156 for the shared failure mode, *wire field edge not block edge*, leading to under‐coverage of the target and confusion over the desired treatment energy (electrons). The largest RPN_QA_ score is similarly shared between the two processes. It relates to the *physicist reviewing the bolus with a misunderstanding of the planned intent*, which can lead to a suboptimal plan or possible medical event. In contrast, the largest RPN value without an associated potential cause of failure *misunderstanding of plan* intent was *importing the incorrect file to meshmixer*. For the creation and fabrication of the BB, exporting the incorrect structures from the TPS and errors in making the silicone mix was the most prominent. Similarly, for the CSB fabrication, the errors in making the silicone mix made up three out of the top five RPN scores. The range of RPN scoring between the two scorers for a single event ranged from 0 to 248, with the maximum score by an individual of 280. The five highest severity scores ranged from 7.5 to 8.5. Four of the five top scores were shared by both processes and not specific to the bolus creation or use, e.g., using the incorrect prescription or modality. The highest severity score directly related to bolus creation was for dosimetry designing the bolus for the wrong site, which could result in a suboptimal plan or medical event.

**TABLE 1:** The 10 highest‐scoring failure modes identified by the FMEA for the bespoke and color sheet bolus workflows.

Rank	Sub‐process^a^	Process step description	Failure mode	Potential cause of failure	O	S	D	D_QA_	RPN	RPN_QA_
1	4 (4)	Wire Treatment Area	Wire field edge, not block edge	Miscommunication, document error, lack of documentation, inattention, misunderstanding of plan intent, training/historical process, verbal orders	3.5	5.5	6	2.5	156	60.5
2	20 (−)	Import STL File to Meshmixer	Import incorrect file	Communication failure, incorrect/lack of documentation, Inattention, carelessness	4.5	5	6.5	2	150	48
3	4 (4)	Wire Treatment Area	Wire incorrect or incomplete region	Miscommunication, document error, lack of documentation, inattention, misunderstanding of plan intent, verbal orders	3	6	6.5	4	137	67
4	15 (20)	Physics Reviews Bolus	Reviews bolus with misunderstanding of plan intent	Communication failure, incorrect/lack of documentation, Inattention, carelessness	3.5	6	7	6	131	103
5	5 (5)	Will a Bronze Shield be Used?	Follow no when should be yes	Inattention, carelessness, lack of training, lack of documentation, incorrect documentation, verbal orders, communication issues	3.5	4	5	1.5	124	32
6	16 (21)	Does Physics Approve the Bolus?	Chooses minor edits but should be major/rejection	Inattention, carelessness, lack of documentation, lack of training, misunderstanding of plan intent	3	6	7	2.5	117	43.5
7	16 (21)	Does Physics Approve the Bolus?	Approves bolus when shouldn't	Inattention, carelessness, lack of documentation, lack of training, misunderstanding of plan intent	3	6	7	2.5	106	43
8	16 (21)	Does Physics Approve the Bolus?	Does neither	Inattention, carelessness, unaware of task, communication failure	3	6	7	2.5	106	43
9	11 (15)	Dosimetry Begins Treatment Plan	Plan on incorrect CT dataset	Select a dataset from a previous course, inattention, carelessness, document error or lack of documentation, communication failure	2.5	8	5	5	102	102
10	12 (17)	Dosimetry Designs Bolus	Use non‐3D workflow when should be 3D	Communication failure, incorrect or lack of documentation, misunderstanding of plan intent	3	5	6	6	96	96

^a^Sub‐process step formatted as BB (CSB).

**TABLE 2:** The five highest‐scoring failure modes identified by the FMEA specific to the bolus creation/fabrication steps for bespoke (top) and the color sheet bolus (bottom).

Rank	Sub‐process^a^	Process step description	Failure mode	Potential cause of failure	O	S	D	D_QA_	RPN	RPN_QA_
1	20 (−)	Import STL File to Meshmixer	Import incorrect file	Communication failure, incorrect/lack of documentation, inattention, carelessness	4.5	5	6.5	2	150	48
2	18 (−)	Export CT & DICOM Structure Set	Make incorrect structures for export	Communication failure, incorrect/lack of documentation, inattention, carelessness	3	4.5	7	2.5	94.5	34.5
3	35 (−)	Make silicone mixture	Incorrect ratio for silicone mixture	Incorrect/lack of documentation, inattention, carelessness	2.5	5	6.5	2.5	82	35
4	18 (−)	Export CT & DICOM Structure Set	Export wrong structures	Communication failure, incorrect/lack of documentation, inattention, carelessness	2.5	4	6	2	62	20
5	39 (−)	Disassemble the mold, retaining the inner shell	Damage bolus during disassembly	Incorrect/lack of documentation, inattention, carelessness	2.5	3.5	3.5	1.5	47	17
1	− (25)	Make silicone mixture	Incorrect ratio for silicone mixture	Incorrect/lack of documentation, inattention, carelessness	2.5	5	6.5	2.5	82	35
2	− (23)	Obtain appropriate colored sheet mold tray	Incorrect tray used	Inattention, carelessness, lack of documentation, lack of training, misunderstanding of plan intent	2	5	4	2	40	20
3	− (25)	Make silicone mixture	Do not add silicone thinner	Communication failure, incorrect/lack of documentation, inattention, carelessness	1.5	5	4.5	1.5	34	11
4	− (25)	Make silicone mixture	Do not degas mixture	Inattention, carelessness, incorrect/lack of documentation	1.5	4.5	4.5	1.5	33	10.5
5	− (26)	Add color corresponding to thickness in question	Add incorrect color	Incorrect or lack of documentation, carelessness, inattention, communication failure	2	4.5	3	1	30	9

^a^Sub‐process step formatted as BB (CSB).

Figures 3, 4, 5 show some unique clinical examples where the 3D‐molded silicone bolus has been used. As mentioned, the H&N region provides some unique challenges that commercially available bolus cannot satisfy. However, we see in Figure 3 that the BB conforms well to the complex anatomy of the nose and eye sockets while simultaneously filling the nasal cavity. Figure 3 also highlights cases where the flexibility of the silicone poured bolus allows it to drape snugly around highly convex surfaces such as the skull for a scalp treatment while also conforming to the ear. Such a result would be unattainable utilizing traditional bolus. The benefits of full implementation are also realized in the thoracic and pelvic regions, as shown in Figures 4 and 5. We see the bolus fitting snugly to the curved surfaces for the inframammary fold and chest wall cases, pulling dose superficially to adequately cover the target volumes. The pelvic cases (Figure 5) highlight more intricate and typically challenging cases where the BB provides a more refined approach. For the vulva case, the bespoke silicone bolus not only conformed exceptionally well to the patient's anatomy, but the extension of the bolus over the legs helped provide feedback for the patient set‐up. Moreover, the rectum case further underscores the adaptability of this in‐house open‐source 3D silicone bolus workflow, where the air gap between the buttocks can be filled reproducibly, more accurately representing the design in the treatment planning system.

## 4 DISCUSSION

Various iterations of mold creation and bolus casting were explored before the currently employed method and workflow were deemed optimal. The desired qualities of the 3D printed mold are easy assembly/disassembly, clearly identifiable parts, and silicone‐tight. The adhesive and sealing properties of the sealant used to adhere the mold together are extremely important. In developing this process, various methods of silicone‐tight, easy‐to‐disassemble seals were tested. Silicone caulk is commonly used in this application. It is reliable and easy to apply but is challenging to remove. Modeling clay cords formed with a pastry extruder showed promise due to ease of application and sealing well on flat surfaces. However, it was challenging to consistently achieve a silicone‐tight seal due in part to its adhesive quality. Ultimately, we determined that the combination of hot glue and spring clamps was a simple and reliable method to assemble and seal the side walls while maintaining ease of disassembly.

**FIGURE 2:**
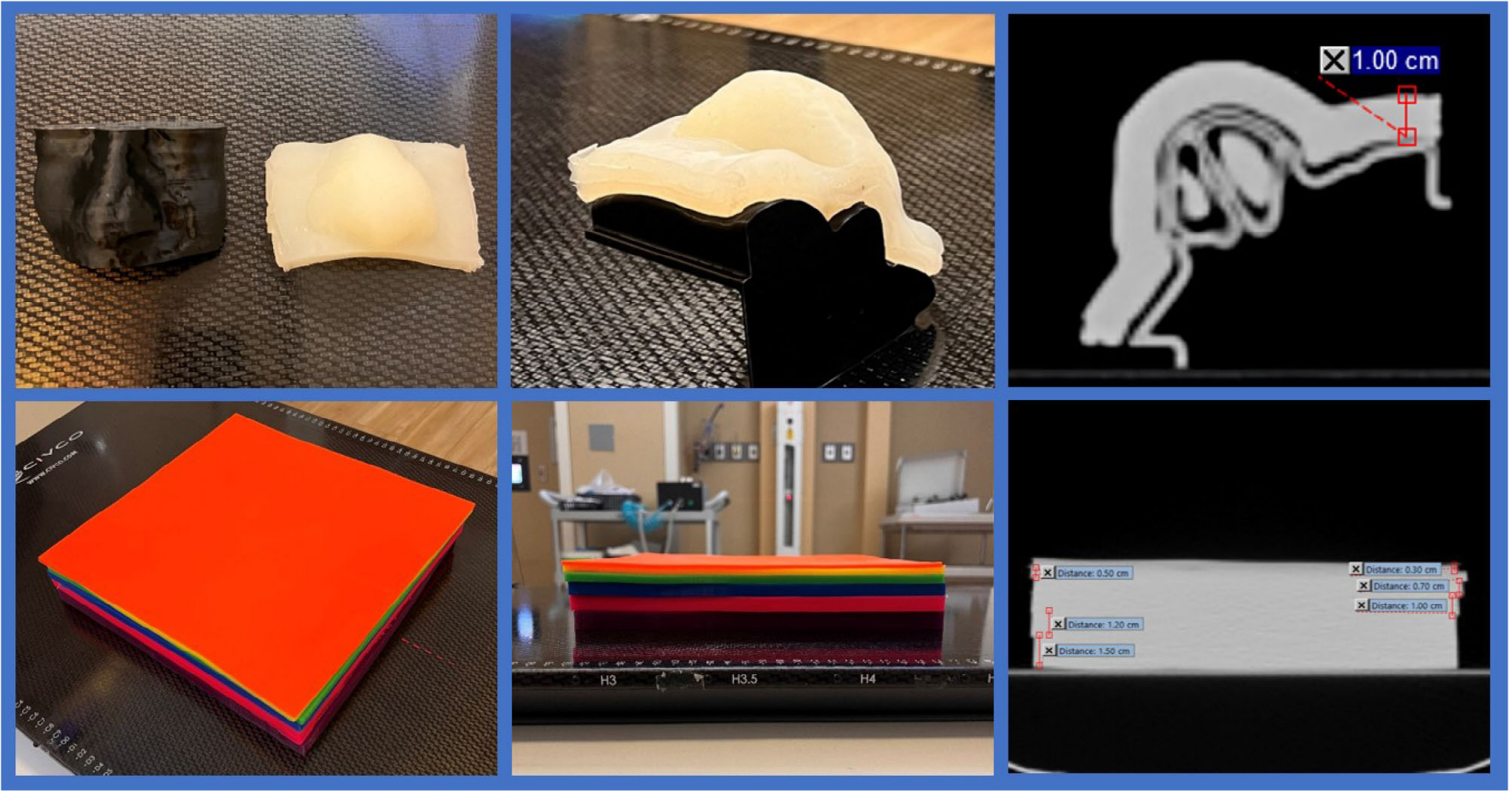
Examples of the bespoke (top) and color sheet (bottom) silicone bolus, and the corresponding QA setup on the CT scanner, with an example of the thickness measurement.

A key component for successfully implementing 3D molded bolus is ensuring the quality of bolus creation. The aforementioned QA tests are deemed comprehensive and reproducible for our purposes. The Shore value provides insight into the ability of the material to drape and accept compressive force. These properties are important for the elimination of air gaps, patient comfort, and conformity to patient anatomy. While the Shore value is not intrinsically vital to the use of 3D molded bolus, it provides a straightforward way to identify gross fabrication errors. Based on the standard deviation of the measured data, a threshold of 17—23 was deemed suitable for patient use. It must be stated that if all other tests pass without issue, an outlier Shore value alone should not disqualify the use of a given bolus. However, this should be investigated further before use. In commercial bolus, a measurement of the exterior edge would suffice in providing a reliable thickness measurement; however, the fundamental principle of the bespoke 3D bolus design in Eclipse (Figure 2) means this approach is inappropriate. Thus, Eclipse's “measure distance” tool was deemed suitable for both the BB and CSB cast bolus, ensuring a consistent measurement normal to the applied surface and a standardized method for both processes. A QA tolerance of ± 1 mm for thickness was established. This value was chosen to balance the dosimetric impact and to minimize false positives due to the resource‐intensive nature of generating a new bolus.

Variations in the mixing protocol can impact the bolus homogeneity, while variation in the CT scan protocol influences the quantity of noise in the scan. Considering the dosimetric data and our clinical goals, any HU variation in the range of 100—200 for QA purposes is deemed acceptable and does not have a concerning dosimetric effect. The CT can also provide insight into hypo‐ and hyper‐dense areas, which could indicate a poorly mixed area or the presence of air bubbles. Throughout the course of the examination, no such regions were found. If any regions of this nature are located in the future, further investigation and evaluation are needed prior to patient use. Furthermore, as the BB is scanned on its 3D‐printed scaffolding (formed from the external contour of the planning CT, Figure S4), the determination of how well the bolus conforms to the intended target area can be investigated. Air gaps should not be present or at least be in an area inconsequential to the dosimetry of the target area. Our evaluations of patient‐specific 3D cast silicone BB showed no cases of poor fitting or air gaps. The dosimetric data justifies the decision to override the HU value of the BB and CSB bolus to 160 HU, standardizing the process for each case. The more considerable discrepancy in the measured dose for the case of 6MV photons compared to 6 MeV electrons is partly attributed to the limitations of the AAA algorithm in the buildup region.

The process map and FMEA for the BB and CSB highlight some similarities between the workflows, with 22 steps common to both processes and 69 shared failure modes. The failure modes with the largest average *RPN* values are shown in Table 1. AAPM's TG100 states that most errors in radiation oncology are due to workflow and processes, which agrees with our FMEA results. Notably, 6 of these 10 identified failure modes center on a lack of understanding or misunderstanding of the planned intent. Thus, physics and dosimetry must be heavily involved at the onset of the process to ensure the physician's intent is clearly understood to provide the highest quality treatment. This led to focused attention on clinical solutions and workflow building to address these highest‐scoring failure modes. The largest *RPN* score for *wire field edge, not block edge*, is an excellent example. The procedure of bolus creation in the TPS is guided by the radiopaque wire placement by the physician at CT simulation. This wire should be placed to define the treatment field edge to design the bolus to provide adequate scatter conditions for coverage. Physician training and experience can play a role in their preference to wire the field or block edge for these treatments, requiring a standardized approach and robust communication for all these cases. Physics and dosimetry presence at CT simulation can begin the conversation about planned intent, potentially reducing the likelihood of occurrence and increasing the detectability of an event. Having physics and dosimetry involvement early in the process would also benefit the failure modes associated with the design and physics approval of the bolus due to the ongoing conversation this allows. Moreover, the FMEA provides insight into an efficient and effective QM program, with the *RPN_QA_* values supplementing the process by pre‐identifying steps where QA may be most impactful.

**FIGURE 3:**
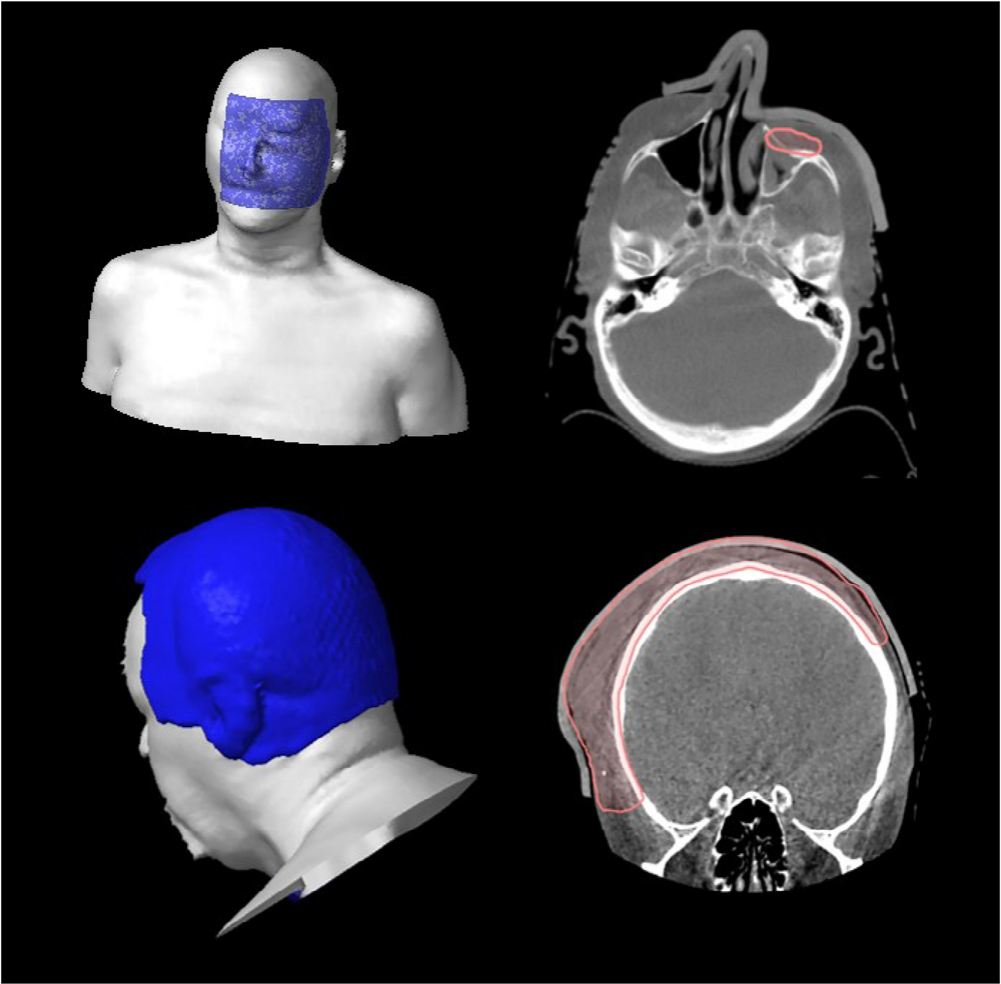
Clinical examples of bespoke silicone bolus use in the head and neck region employed to conform to the complex anatomy of the face while filling the nasal cavity (top) and to conform to convex surface of skull and anatomy of the ear (bottom).

**FIGURE 4:**
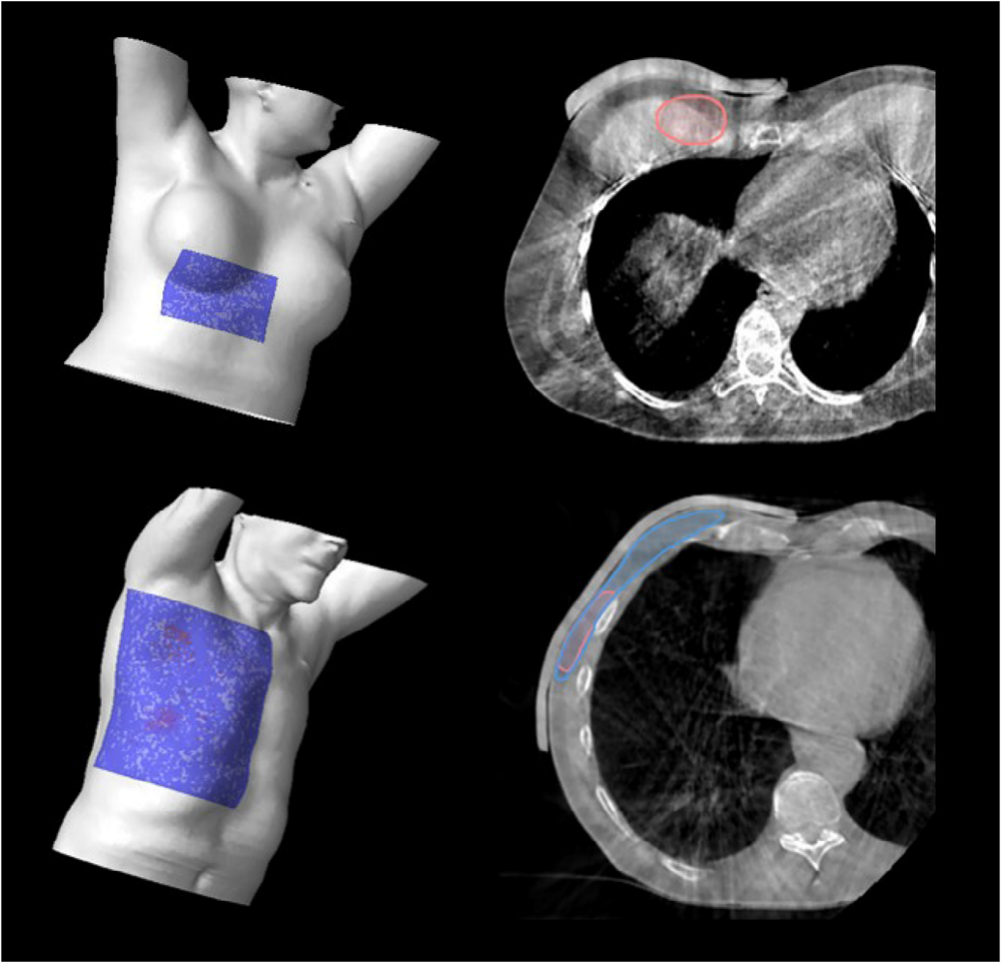
Clinical examples of bespoke silicone bolus use in the thorax region employed to shape around the inframammary fold (top) and conform to the convex surface of a chest wall (bottom).

**FIGURE 5:**
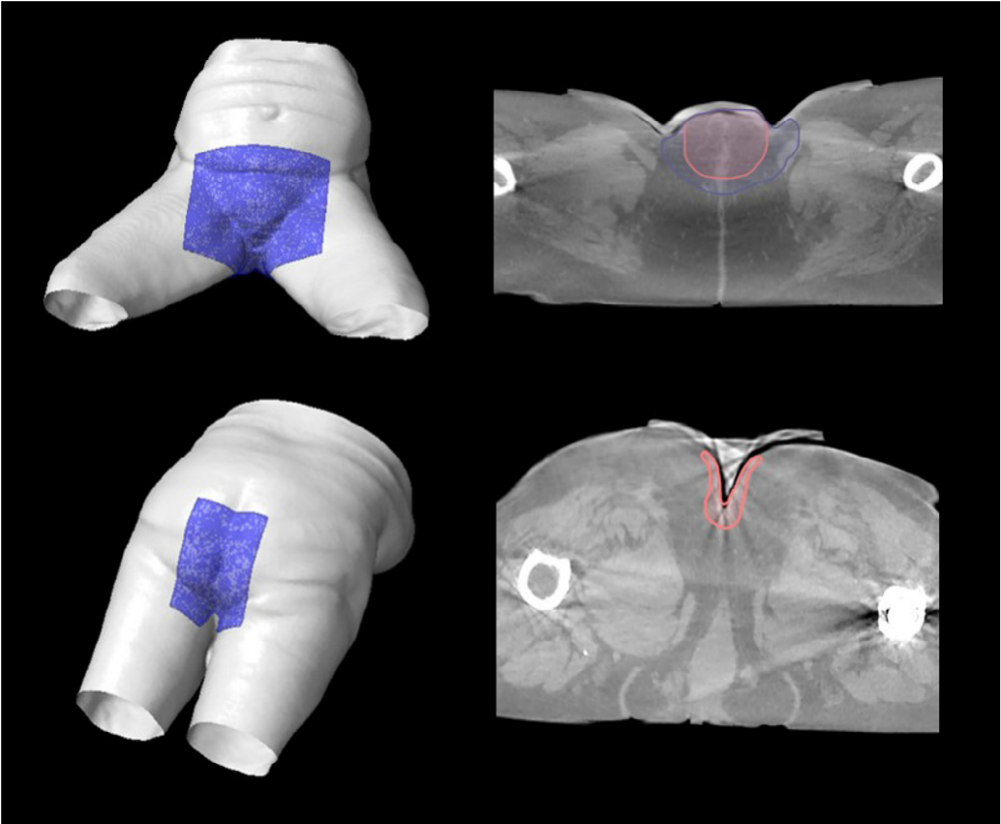
Clinical examples of bespoke silicone bolus use in the pelvic region employed for conformity to vulva anatomy (top) and to fill air gaps between buttocks (bottom).

The fundamental cause for severe failures in this process from the FMEA is the misunderstanding of plan intent, which can occur in multiple instances in the treatment process, necessitating QA/QC steps in addition to those focused on the fabrication of the bolus. Standardized nomenclature for the different bolus was implemented to ensure that anyone looking at the plan can immediately identify the type of bolus used and the corresponding standard operating procedure, minimizing verbal miscommunications. Moreover, periodic training and education of the various teams and in‐depth documentation provide additional layers of safety for the different bolus processes and give team members the confidence to speak up if they see a potential issue arise. The approaches described above also benefit the highest‐scoring RPN values in Table 2 for the BB process, allowing for possible automation. A unique QC safeguard was implemented for the CSB, where the molds for the different thicknesses were color‐coded (Figure S4) to match the color of the resulting boluses. Such a safeguard reduces the RPN value for two of the top five highest‐scoring failure modes for the CSB bolus (Table 2). The color coding of the molds and bolus is designed as an extra safety net and does not negate the physical measurement of bolus thicknesses. Any discrepancy in the color of the bolus used, or the thickness measurement should elicit a response to ensure the fidelity of the planned treatment intent. This color coding can highlight issues at simulation and on the day of treatment for the CSB. However, the same approach could not be used for the BB. The BB bolus is not present at simulation and is virtually added in the planning process. Thus, the safety guards are implemented post‐fabrication (i.e., QA) and at the first treatment fraction. Physics presence is required at the first fraction to ensure the treatment is executed with the utmost quality and fidelity. A reference field where the multi‐leaf collimators are shaped to the bolus contour is projected onto the patient using the light field to confirm the correct bolus is being utilized, as well as to assist the therapists with initial set‐up and placement. Upon initial placement, a CBCT image is acquired for additional assessment of bolus placement, such as possible air gaps and filling of cavities, which are not always visible with the in‐room inspection. Examples of the first fraction of CBCTs can be observed in Figures 3, 4, 5. The light reference field was often sufficient for a high‐quality setup with minimal air gaps. However, some cases with more complex surfaces and/or cavity filling benefited from the CBCT, allowing for minor adjustments to obtain the most accurate setup. Initially, a core team of physicists trained in the bolus process would cover the first fraction. Once the implementation was more mature, adequate training and documentation were provided, and the duty was transferred to the physicist of the day once the team was comfortable.

A high‐level approach was also taken to mitigate the impact of the failure modes by splitting the clinical rollout into multiple phases: phase 1, uniform thickness bolus for electrons and photons; phase 2, non‐uniform thickness bolus for photons; phase 3, non‐uniform thickness bolus for electrons; phase 4, unique and challenging cavity filling. Furthermore, sub‐phases allowed for a safe and high‐quality 3D molded bolus program rollout. The sub‐phases for phase 1 implementation were: 1a, introduction, familiarization, and workflow refinement; 1b, expansion to cutting thermoplastic mask for use; 1c, expansion to non‐H&N sites; 1d, expansion to use under masks without cutting (direct use of sheets as SF replacement); 1e, expansion to use with 3D printed bronze on‐skin shielding. This is partly due to the ability to focus the training into more manageable chunks, preventing fatigue and the feeling of being overwhelmed, which helps maintain buy‐in from each team for the entirety of the project implementation. Progression from each phase depended upon the success of the implementation as deemed by each team with assessment following the treatment of a specified number of patients. This allowed the ability to fine‐tune the steps at each phase and discuss any withstanding issues that must be addressed to ensure the program's continued success. Feedback from each team for their perspective is crucial for a comprehensive analysis of the implemented phase and garnering support for the following phases.

## 5 CONCLUSION

In this study, we describe the virtual elimination of commercial bolus in our clinic using bespoke and flat sheet silicone bolus molded in‐house from 3D printed casts. A comprehensive failure modes and effects analysis assessed each workflow process, identifying the highest scoring and most severe failure modes. This informed the design and implementation of quality assurance steps to guarantee safe and high‐quality treatments. The bespoke boluses allow for greater adaptability not afforded by traditional commercial boluses, such as cavity filling and improved conformity to complex surfaces. Moreover, the approach presented here uses open‐source or free software, making it accessible to any practice with access to a 3D printer.

## AUTHOR CONTRIBUTIONS

Contributed to the design, acquisition, analysis, and interpretation of the work: Courtney R. Buckey, Michael D. Armstrong, Yi Rong, and Dean Hobbis. *Contributed to data acquisition, analysis, or interpretation*: Samir H. Patel, Riley C. Tegtmeier, Brady S. Laughlin, Shadi Chitsazzadeh, Edward L. Clouser, Jennifer L. Smetanick, Justin Pettit, Justin D. Gagneur, and Joshua B. Stoker. All authors drafted or critically revised the work for its intellectual content and agree to be accountable for all aspects of the work presented.

## CONFLICT OF INTEREST STATEMENT

All authors have no relevant financial interests to be declared.

## Supporting information

Supporting Information

## References

[1] Rooney MK , Rosenberg DM , Braunstein S , et al. Three‐dimensional printing in radiation oncology: a systematic review of the literature. J Appl Clin Med Phys. 2020;21(8):15‐26. 10.1002/acm2.12907 PMC7484837 32459059

[2] Sakin M , Kroglu YC . 3D printing of buildings: construction of the sustainable houses of the future by BIM. Energy Procedia. 2017;134:702‐711.

[3] Tino R , Leary M , Yeo A , et al. Additive manufacturing in radiation oncology: a review of clinical practice, emerging trends and research opportunities. Int J Exrem Manuf. 2020;2:012003.

[4] Chuan YF , Hong NL , Mahdi MA , et al. Three‐dimensional printed electrode and its novel applications in electronic devices. Sci Rep. 2018;1:11‐18. 10.1038/s41598-018-25861-3 PMC5943534 29743664

[5] Joshi SC , Sheikh JJ . 3D‐printing in aerospace and its long‐term sustainability. Virtual Phys Prototyp. 2015;10:175‐185.

[6] Paul GM , Rezaienaia A , Wen P , et al. Medical applications for 3D printing: recent developments. Mo Med. 2018;115:75‐81. 30228688 PMC6139809

[7] Sreehitha V . Impact of 3D printing in automotive industry. Int J Mech Prod Eng. 2017;5:91‐94.

[8] Welch R , Hobbis D , Birnbaum AJ , et al. Nano‐ and micro‐structures formed during laser processing of selenium doped bismuth telluride. Adv Mater Interfaces. 2021;8(15):2100185.

[9] Zhang H , Hobbis D , Nolas GS , et al. Laser additive manufacturing of powdered bismuth telluride. J Mater Res. 2018;33:4031‐4039.

[10] Craft DF , Howell RM . Preparation and fabrication of a full‐scale, sagittal‐sliced, 3D‐printed, patient‐specific radiotherapy phantom. J Appl Clin Med Phys. 2017;18(5):285‐292. 28857407 10.1002/acm2.12162 PMC5874860

[11] Arenas M , Sabater S , Sintas A , et al. Individualized 3D scanning and printing for non‐melanoma skin cancer brachytherapy: a financial study for its integration into clinical workflow. J Contemp Brachytherapy. 2017;9:270‐276. 28725252 10.5114/jcb.2017.68134 PMC5509979

[12] Lindegaard JC , Madsen ML , Traberg A , et al. Individualised 3D printed vaginal template for MRI guided brachytherapy in locally advanced cervical cancer. Radiat Oncol. 2016;118:173‐175. 10.1016/j.radonc.2015.12.012 26743833

[13] Arenas M , Sabater S , Sintas A , et al. Individualized 3D scanning and printing for non‐melanoma skin cancer brachytherapy: a financial study for its integration into clinical workflow. J Contemp Brachytherapy. 2017;9(3):270‐276. 28725252 10.5114/jcb.2017.68134 PMC5509979

[14] Lindegaard JC , Madsen ML , Traberg A , et al. Individualised 3D printed vaginal template for MRI guided brachytherapy in locally advanced cervical cancer. Radiother Oncol. 2016;118(1):173‐175. 26743833 10.1016/j.radonc.2015.12.012

[15] Meyer T , Quirk S , D'Souza M , et al. A framework for clinical commissioning of 3D‐printed patient support or immobilization devices in photon radiotherapy. J Appl Clin Med Phys. 2018;19:499‐505. 29984551 10.1002/acm2.12408 PMC6123103

[16] Haefner MF , Giesel FL , Mattke M , et al. 3D‐printed masks as a new apporach for immobilzation in radiotherapy—a study of positioning accuracy. Oncotarget. 2018;9:6490‐6498. 29464087 10.18632/oncotarget.24032 PMC5814227

[17] Asfia A , Novak JI , Mohammed MI , et al. A review of 3D printed patient specific immobilisation devices in radiotherapy. Phys Imaging Radiat Oncol. 2020;13:30‐35. 33458304 10.1016/j.phro.2020.03.003 PMC7807671

[18] Mattke M , Rath D , Hafner MF , et al. Individual 3D‐printed fixation masks for radiotherapy: first clinical experiences. Int J Comput Assist Radiol Surg. 2021;16(6):1043‐1049. 34021859 10.1007/s11548-021-02393-2 PMC8166668

[19] Baltz GC , Chi PM , Wong PF , et al. Development and validation of a 3D‐printed bolus cap for total scalp irradiation. J Appl Clin Med Phys. 2019;20(3):89‐96. 10.1002/acm2.12552 PMC6414136 30821903

[20] Burleson S , Baker J , Hsia AT , et al. Use of 3D printers to create a patient‐specific 3D bolus for external beam. J Appl Clin Med Phys. 2015;16:166‐178. 10.1120/jacmp.v16i3.5247 PMC5690114 26103485

[21] Chiu T , Tan J , Brenner M , et al. Three‐dimensional printer‐aided casting of soft, custom silicone boluses (SCSBs) for head and neck radiation therapy. Pract Radiat Oncol. 2018;8(3):e167‐e174. 29452869 10.1016/j.prro.2017.11.001

[22] Craft DF , Balter P , Woodward W , et al. Design, fabrication, and validation of patient‐specific electron tissue compensators for postmastectomy radiation therapy. Phys Imaging Radiat Oncol. 2018;8:38‐43. 33458415 10.1016/j.phro.2018.11.005 PMC7807570

[23] Kim SW , Shin HJ , Kay CS , et al. A customized bolus produced using a 3‐dimensional printer for radiotherapy. PLoS One. 2014;9(10):e110746. 25337700 10.1371/journal.pone.0110746 PMC4206462

[24] Lukowiak M , Jezierska K , Boehlke M , et al. Utilization of a 3D printer to fabricate boluses used for electron therapy of skin lesions of the eye canthi. J Appl Clin Med Phys. 2017;18(1):76‐81. 10.1002/acm2.12013 PMC5689892 28291910

[25] Park K , Park S , Jeon M‐J , et al. Clinical application of 3D‐printed‐step‐bolus in post‐total‐mastectomy electron conformal therapy. Oncotarget. 2017;8:25660‐25668. 27784001 10.18632/oncotarget.12829 PMC5421959

[26] Wang KM , Rickards AJ , Bingham T , et al. Technical note: evaluation of a silicone‐based custom bolus for radiation therapy of a superficial pelvic tumor. J Appl Clin Med Phys. 2022;23(4):e13538. 35084098 10.1002/acm2.13538 PMC8992939

[27] Butson MJ , Cheung T , Yu P , et al. Effects on skin dose from unwanted air gaps under bolus in photon beam radiotherapy. Radiat Meas. 2000;32:201‐204.

[28] Khan Y , Villarreal‐Barajas JE , Udowicz M , et al. Clinical and dosimetric implications of air gaps between bolus and skin surface during radiation therapy. J Cancer Ther. 2013;4:1251‐1255.

[29] Kong M , Holloway L . An investigation of central axis depth dose distribution perturbation due to an air gap between patient and bolus for electron beams. Australas Phys Eng Sci Med. 2007;30:111‐119. 17682400 10.1007/BF03178415

[30] Shahrubudin N , Lee TC , Ramlan R . An overview of 3D printing technology: technological, materials, and applications. Procedia Manuf. 2019;35:1289‐1296.

[31] Park JW , Oh SA , Yea JW , et al. Fabrication of malleable three‐dimensional‐printed customized bolus using three‐dimensional scanner. PLoS One. 2017;12(5):e0177562. 28494012 10.1371/journal.pone.0177562 PMC5426771

[32] Canters RA , Lips IM , Wendling M , et al. Clinical implementation of 3D printing in the construction of patient specific bolus for electron beam radiotherapy for non‐melanoma skin cancer. Radiother Oncol. 2016;121(1):148‐153. 27475278 10.1016/j.radonc.2016.07.011

[33] Ehler E , Sterling D , Dusenbery K , et al. Workload implications for clinic workflow with implementation of three‐dimensional printed customized bolus for radiation therapy: a pilot study. PLoS One. 2018;13(10):e0204944. 30273403 10.1371/journal.pone.0204944 PMC6166970

[34] Huq S , Fraass BA , Dunscombe PB , et al. The report of Task Group 100 of the AAPM: application of risk analysis methods to radiation therapy quality management. Med Phys. 2016;43(7):4209‐4260. 27370140 10.1118/1.4947547 PMC4985013

[35] Test No. 439: In Vitro Skin Irritation: Reconstructed Human Epidermis Test Method, OECD Guidelines for the Testing of Chemicals, Section 4. OECD Publishing; 2020.

[36] Zhao H , Allanson D , Ren J . Use of shore hardness tests for in‐process properties estimation/monitoring of silicone rubbers. J Mater Sci Chem Eng. 2015;3(7).

