# Supplementary material for: Comprehensive clinical implementation, workflow, and FMEA of bespoke silicone bolus cast from 3D printed molds using open‐source resources
Source: J Appl Clin Med Phys. 2024 Aug 27;25(11):e14498. doi: 10.1002/acm2.14498 (PMC11539970; doi:10.1002/acm2.14498)
Supplement: Supplementary file 1 — Supporting Information [file ACM2-25-e14498-s001.docx]

Bolus Mold Workflow

Overview

The bolus mold demonstrated here is a five-piece set that consists of inner and outer shells, matching side walls, and a bottom. This basic technique is best suited to a rectangular-shaped bolus but can be adapted to other shapes.

The DICOM CT and structure set are imported into 3D Slicer for the purpose of saving the bolus as a stl file. Mold design is done in Meshmixer, and the mold parts are then printed on one or more 3D printers. The parts are then hot melt glued together, and small spring clamps secure the walls to the shells to ensure mold integrity. The mold is then poured with thinned and degassed Ecoflex 00-30 silicone. Demolding is a matter of removing the spring clamps and removing the walls. The inner shell is used as a carrier for the new bolus.

The silicone referenced is Smooth-On Ecoflex 00-30 that was mixed 50/50 from parts A and B, thinned 10% by mass with Smooth-on silicone thinner and thoroughly degassed in a vacuum tank until the bubbles subside. Degassing is critical for a defect free bolus.

Workflow

1. Import the CT and structure set into 3D Slicer.
2. In the Segmentations module, select Export and Models and then click on Export.
3. Select Save Data and select only the bolus. Then select stl as the extension.
4. Import the stl file into Meshmixer. Select Edit on the left side of the screen and press Make Solid.
5. Select Analysis/Stability on the left of the screen and record the Volume of the bolus.
6. Click on the Meshmix button on the left and drag a cube into the scene.
7. Make the cube 15mm thick and size the other dimensions to form a wall on one side of the bolus, extending from top to bottom with space on front and back of the bolus large enough so small spring clamps can pinch it. Add a wall to the other side in the same way. The walls serve two purposes: to keep the silicone in and to set the spacing to the designed bolus thickness. It is desirable to make both side walls the same size.
8. Add another cube and set the thickness to 15mm and adjust the other two dimensions to the footprint of the bolus as looking down from above.
9. Select all parts in Object Browser and click Export.
10. With the five parts still selected, click Combine and then Make Solid.
11. There is now one solid piece. Click Select at the left of the screen and double click the model. The model will turn orange when selected.
12. Select Edit…/Offset and set Distance to 3mm. This will be the wall thickness of the mold parts.
13. Ensure the entire model is still selected and click Modify…/Invert and then Edit…/Flip Normals. This properly orients the insides and outsides. If there is a pink wavy surface, the normals are reversed.
14. Use the plane cut tool to cut through half the thickness of the side walls (about 7.5mm).
15. Use the plane cut tool to cut through the thickness of the bottom wall so it will lie flat on the print bed without requiring support structure underneath.
16. Now plane cut the top of the mold until the opening is large enough to easily pour the silicone inside. At this point the inner and outer shells should be separate.
17. With nothing selected, click Edit on the left of the screen and then Make Solid. Then click Select and double click the outer shell to select it. Delete it temporarily and export the inner shell. Ctrl-Z to undo deleting the outer shell and export the inner shell in the same way.
18. Reload the file with all parts that was previously exported.
19. Change the thickness of the wall and bottom to 3mm. Select only the wall, and export. Export the bottom in the same way.
20. Print the two shells, bottom and two walls. The walls should be the same size. (see Figure S1)
21. Apply a generous bead of hot melt glue on both sides of the shell near the cavity opening and attach the wall, pushing the shell sides together toward the wall. Apply spring clamps to hold the walls in place. The hot melt glue in this case is both a sealant and a weak adhesive, so adhesion is augmented by the clamps. With this method, the mold can be poured immediately after assembly. Remember that the walls also set the bolus thickness, so make sure the walls fit against the shell flange (see figure S2).
22. Adhere and seal the bottom and place a heavy flat object on top of the mold to apply downward pressure until the glue cools. This should take a few minutes. Fill the mold with the silicone mixture.
23. After the four-hour cure time for Ecoflex 00-30, disassemble the mold. Retain the inner shell as the bolus carrier (see figure S3).


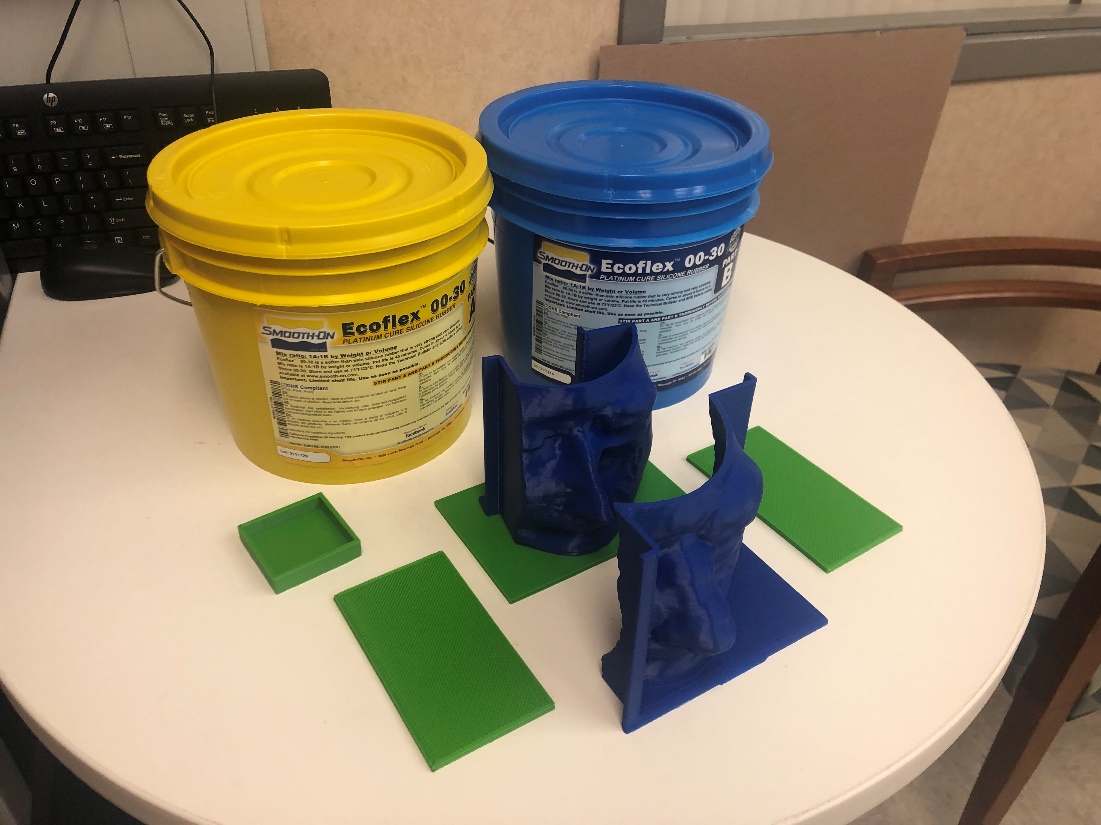
Figure S1. Example mold (blue) for bespoke H&N bolus with walls to secure (green).


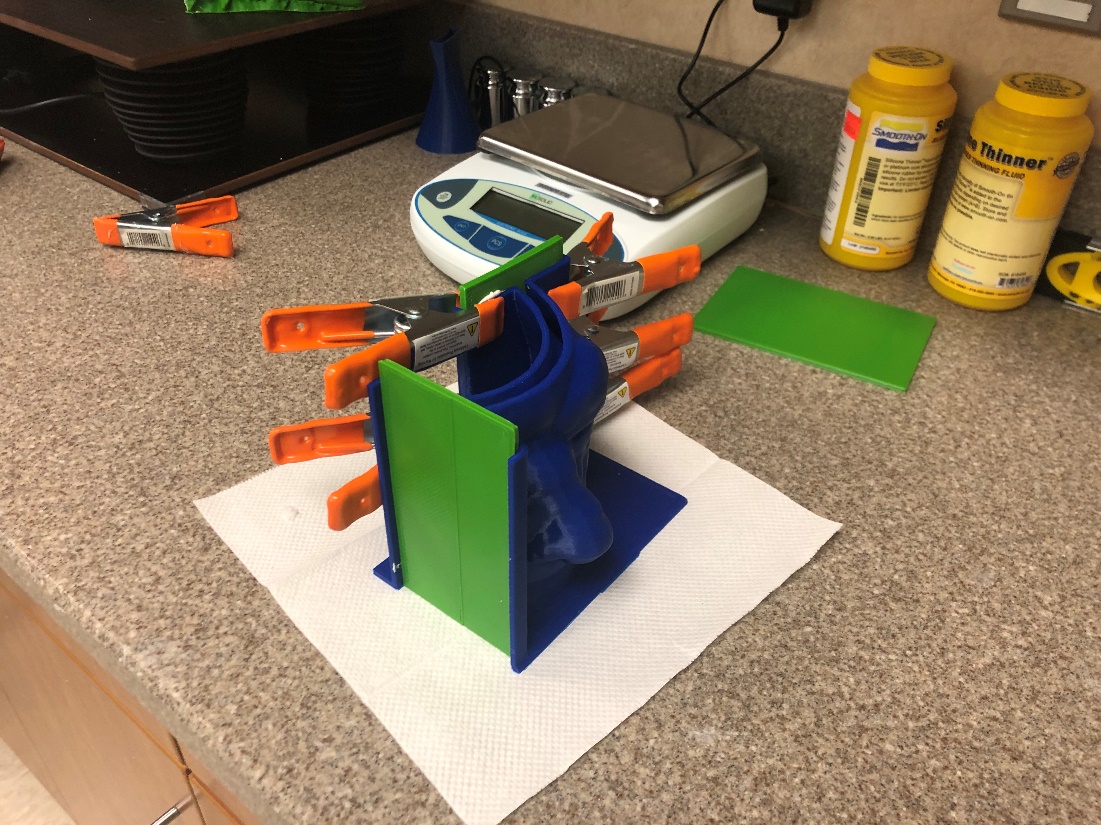
Figure S2. Mold fully assembled with hot glue gun and spring clamps. Ready for pouring the silicone


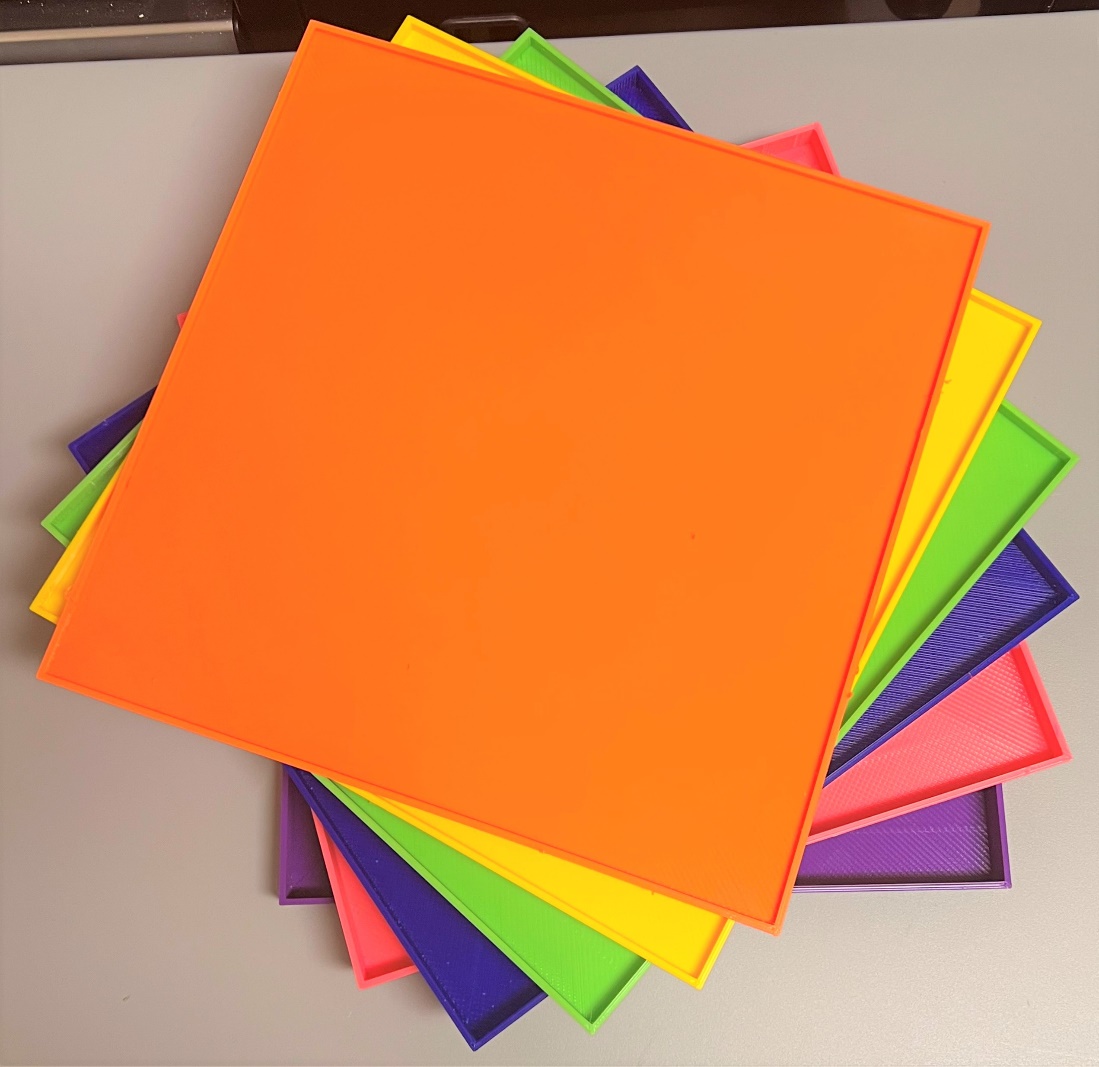
Figure S3. Color-coded mold trays for the five different thicknesses (0.3, 0.5, 0.7, 1.0, 1.2, & 1.5 cm).


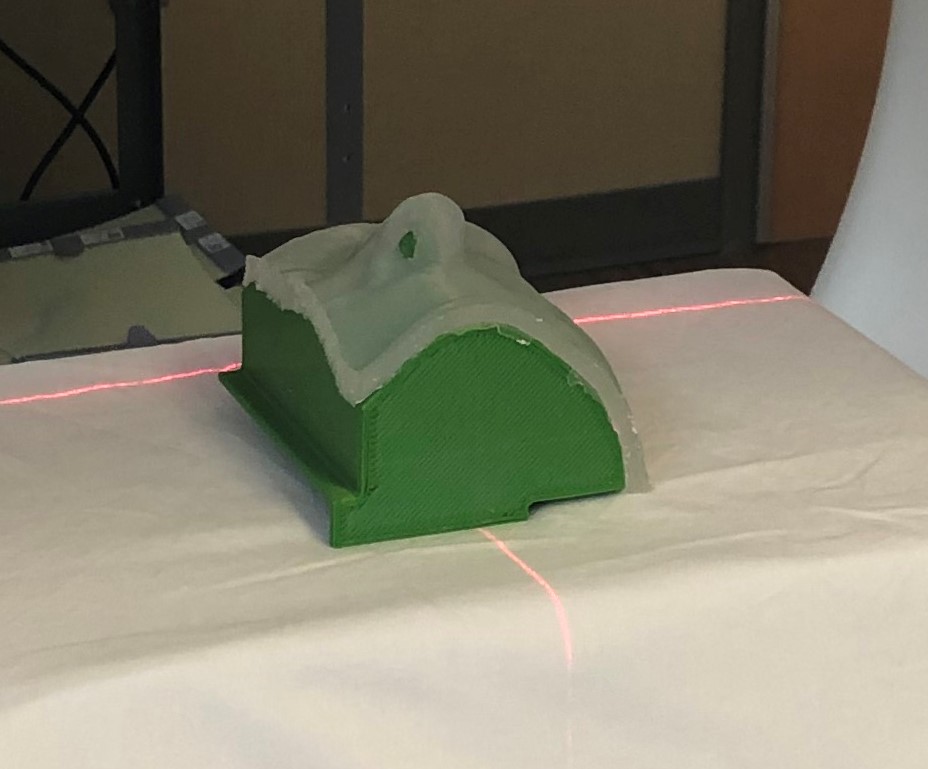
Figure S4. Bespoke H&N bolus on its inner scaffolding, ready for a QA scan on the CT simulator
